# Seasonal Variation, Fractional Isolation and Nanoencapsulation of Antioxidant Compounds of Indian Blackberry (*Syzygium cumini*) †

**DOI:** 10.3390/antiox10121900

**Published:** 2021-11-26

**Authors:** Rabia Shaheen, Muhammad Asif Hanif, Shafaq Nisar, Umer Rashid, Zubia Sajid, Muhammad Raffi Shehzad, Jill K. Winkler-Moser, Ali Alsalme

**Affiliations:** 1 Nano and Biomaterials Lab, Department of Chemistry, University of Agriculture, Faislabad 38040, Pakistan; 2015ag2263@uaf.edu.pk (R.S.); 2014ag1464@uaf.edu.pk (S.N.); 2017ag3270@uaf.edu.pk (Z.S.); 2015ag1523@uaf.edu.pk (M.R.S.); 2 Institute of Nanoscience and Nanotechnology (ION2), Universiti Putra Malaysia, Serdang 43400, Selangor, Malaysia; 3 United States Department of Agriculture, Agricultural Research Service, Peoria, IL 61604, USA; jill.moser@usda.gov; 4 Chemistry Department, College of Science, King Saud University, Riyadh 1145, Saudi Arabia; aalsalme@ksu.edu.sa

**Keywords:** Indian blackberry, essential oil, fractions, bioactivates, nanoparticles

## Abstract

Indian blackberry (*Syzygium cumini* L.) is an evergreen tree in the Myrtaceae family. It is used in traditional medicine due to its significant bioactivities and presence of polyphenols with antioxidant activities. The present study describes the effect of seasonal variations on Indian blackberry leaf essential oil yield and chemical composition, production of fractions from essential oil using high vacuum fractional distillation and slow cooling to low temperature (−50 °C) under vacuum, and bioactivities of the essential oil, fractions, and nanoparticles. The results show that Indian blackberry essential oil yield was higher in spring season as compared to winter season. Indian blackberry essential oil fractionation processes were effective in separating and concentrating compounds with desired bioactivities. The bioactivities shown by magnesium nanoparticles were comparatively higher than barium nanoparticles.

## 1. Introduction

Plants have been used for medicinal purposes since time immemorial and these traditional medicines are still popular worldwide due to lower cost and side effects compared to conventional medicine [1]. Indian blackberry (*Syzygium cumini*), commonly known as Jamun, is a tree belonging to the plant family Myrtaceae. Its leaves have high concentrations of polyphenols and other bioactive compounds effective in treating various ailments in the traditional system of medicine, the Ayurveda [2]. Indian blackberry is an evergreen plant that is cultivated in Pakistan, India, Burma, Nepal, Indonesia, and Sri Lanka. Its leaves are 6 to 12 cm in length and are of variable shape with a smooth shiny surface [3]. The plant produces clusters of oblong fruits that each contain one large seed. The fruit is described as sweet, sour, and astringent and is widely consumed. The seed, fruit pulp, and leaves are used in traditional medicine to treat a wide range of conditions including gastric disorders, leucorrhea, diabetes, fever, hemorrhoids, wounds, and skin disorders [4]. The leaves, fruit, bark, and seeds contain phytochemicals including alkaloids, anthraquinones, flavonoids, catechins, glycosides, phenols, steroids, saponins, tannins, and cardiac glycosides [4]. The diverse bioactivities may be due to antioxidant and free radical scavenging ability of these phytochemical [5].

Essential oils (EO) are concentrated liquids containing mixtures of volatile hydrophobic compounds that retain the natural aroma of their source. EO often have stronger aromas and bioactivities than their sources due to higher concentrations of active ingredients. EO can be extracted from various plant tissues including leaves, bark, stem, roots, fruits, flowers, and peel. Major constituents of the EO are terpenoids that may be monoterpenes or sesquiterpenes, and aromatic moieties such as aldehydes, alcohols, phenols, and methoxy derivatives. EO are often used for medicinal purposes [6] and as flavors and fragrances. Essential oils often have strong antioxidant activities due to free radical scavenging properties [7].

The fractional distillation process is used to purify or isolate compounds of interest from EO based on their boiling points [8]. Nanoencapsulation is the process of capturing substances in various coating materials at the nanoscale range. This technique is particularly useful for application of bioactive materials in foods, cosmetics, and pharmaceuticals. The most common reasons for the use of nanoencapsulation are to protect components from loss of bioactivity from oxidation or thermal degradation, to reduce loss of volatiles, and for targeted drug delivery [9].

As described above, phytochemicals present in various parts of Indian blackberry had significant antioxidant activity [10,11]. Various extracts of Indian blackberry were also observed to have potential to be used as green drug for the treatment of several infections and diseases. However, natural products have some drawbacks in terms of their complex chemistry, access and supply, bioavailability issues, and time-consuming action [12]. Natural products bioavailability has been a major area of concern. Although there are other alternative strategies available, each with its own benefits and drawbacks, one revolutionary strategy among them is nanotechnology [13]. With the flaws in the current delivery systems, a change to nano delivery is urgently required. Nanoparticle-based systems have gotten a lot of attention because of their size, penetration, and many other advantages [14].

Pakistan is one of the major growers of Indian blackberry. Indian blackberry leaves have been extensively used in traditional medicine. However, the composition of bioactive compounds is not fully understood. In the present study, the seasonal variation in yield and composition of Indian blackberry EO was examined. The oil was fractionated, and magnesium (Mg) and barium (Ba) nanoparticles were prepared to evaluate the effect of the nano-encapsulation on bioactive properties, including antioxidant properties.

## 2. Materials and Methods

### 2.1. Materials

Indian blackberry leaves (100 kg) were collected at random from thirty different trees grown at the University of Agriculture, Faisalabad, Pakistan, during the winter and spring seasons. The collected leaves were washed with water to remove debris and dust.

### 2.2. Extraction and Fractionation of Essential Oil

EO was extracted from the leaves using pilot scale hydro-distillation [15]. The obtained EO was separated from water using a separatory funnel and stored at 4 °C for further analysis. Two different processes were used to isolate fractions from the EO. In the first, high vacuum (−760 mmHg) fractional distillation was used to isolate three different constant boiling fractions along with the residue [8]. In the second, EO was slowly cooled to −50 °C using a lyophilizer (model BL-10B, Bioland, Shaanxi, China) to isolate two fractions (crystalline and non-crystalline fractions). For this purpose, the EO was loaded into a petri dish and placed into the lyophilizer for 48 h. Sample code descriptions are provided in Table 1.

### 2.3. Synthesis of Nanoparticles

**Figure 1:**
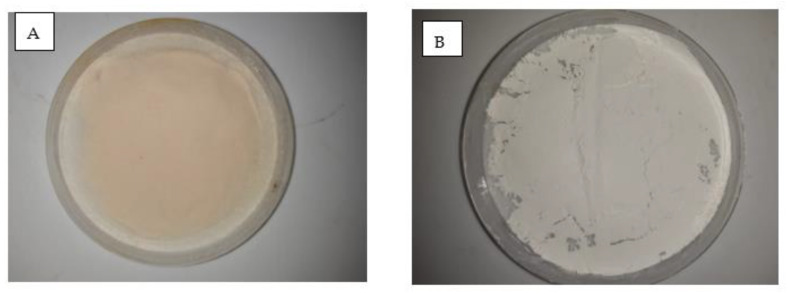
Indian blackberry essential oil nanoparticles (**A**) Ba (**B**) Mg.

Magnesium oxide nanoparticles of EO and EO fractions were prepared using magnesium nitrate by an oxidation process [16]. One gram of EO was added under constant stirring to 25 mL methanol and 20 mL distilled water heated to 60 °C. Then the temperature was increased to 80 °C and 5 g magnesium nitrate was added. After 5 min, the reaction mixture changed color from yellow to brownish yellow as an indication of synthesis of nanoparticles (Figure 1). After filtration, a solid mass of nanoparticles was obtained. Barium sulfate nanoparticles were prepared from barium chloride through a precipitation process [17]. A saturated solution of barium chloride was prepared in 100 mL of distilled water. The barium chloride solution was heated to 60 °C followed by the addition of 1 g of EO or EO fractions. Under constant stirring, 5 mL of saturated anhydrous sodium sulfate solution was added dropwise. A white precipitate settled down in the beaker and was separated out through filtration and completely dried at 60 °C [9,18].

### 2.4. Determination of Total Phenolics, Flavonoids, and Free Radical Scavenging Activities

Total phenolic contents (TPC) of Indian blackberry isolates were determined using Folin–Ciocalteu reagent. To 1 mL of EO, EO fractions, EO nanoparticles (0.5 g of EO nanoparticles dissolved in 1 mL of methanol) and EO fraction nanoparticles (0.5 g of EO fraction nanoparticles dissolved in 1 mL of methanol), 5 mL of Folin–Ciocalteu reagent and 4 mL saturated sodium carbonate was added and mixed by shaking. The mixture was left in the dark at room temperature for 30 min, after which the absorbance was read at 765 nm. TPC was expressed in the gallic acid equivalent in milligrams per gram of EO or EO fraction using a standard curve of gallic acid ranging from 20 to 100 mg/L [19].

Total flavonoid contents were determined using an aluminum trichloride colorimetric assay using catechin [20] as a standard at five concentrations: 2, 40, 60, 80, and 100 mg/L. One ml of EO or EO fractions were added to a beaker containing 4 mL of distilled water and 0.3 mL of 5% NaNO_2_. After 5 min shaking, 0.3 mL of AlCl_3_ and 2 mL of 1 M NaOH were added and after complete mixing the absorbance was measured at 510 nm.

The free radical scavenging potential of EO and EO fractions and nanoparticles was measured using the stable free radical, 1,1-diphenyl-2-picryl-hydrazyl (DPPH) [21,22]. DPPH (0.09 mM) was prepared in 95% MeOH in 100 mL volumetric flask and held in the absence of light for 2 h to stabilize its absorbance. To 2.5 mL of EO or EO fractions, 1 mL of 0.09 mM DPPH solution was mixed, and total volume was made up to 4 mL using 95% MeOH. Absorbance was read at 515 nm and butylated hydroxyl toluene (BHT) (100 ppm), a synthetic antioxidant, was used as a positive control.

### 2.5. Gas Chromatographic Mass Spectrometric (GC-MS) Analysis

After extraction, moisture was removed from the EO using anhydrous sodium sulphate, then filtered prior to injection to the GC-MS. Components were separated following the method described by Ayub et al. [22] using Agilent-Technologies 6890N GC equipped with 7890A series inert XL mass selective detector and 5975C series auto injector (Agilent-Technologies, Santa Clara, CA, USA) along with DB-5 capillary column (50 m × 0.25 mm, film thickness of 0.25 μm). The National Institute of Standards and Technology (NIST) mass spectral library was used for the positive identification of compounds which were later confirmed using commercial standards.

### 2.6. Statistical Analysis

The significance of the obtained result has been tested for ANOVA using SPSS software, whereas Duncan’s test was used for mean comparison at a significance level of 0.05 (*p* ≤ 0.05). Each experiment was done in triplicate and reported as mean ± SD.

## 3. Results and Discussion

### 3.1. Seasonal Variations in Essential Oil Yield and Composition

EO are industrially important natural products of aromatic plants [23]. It is well known that EO yield significantly varies with the season [24,25,26]. Indian blackberry leaf EO was obtained during winter and spring seasons. Indian blackberry leaf EO yield was 0.48 ± 0.11% and 0.62 ± 0.14%, respectively, during the winter and spring seasons. The reason behind this might be more favorable growth conditions during the spring season [27]. Environmental factors have a significant effect on the physiological regulation of metabolic and biochemical processes that regulate carbon flow as well as on the turnover rates of terpenoid/phenylpropanoid compound [28]. A significant effect of harvesting season on EO yield has also been found in previous studies [28,29,30,31].

A total of 52 and 45 compounds were identified in Indian blackberry leaf EO harvested in spring and winter seasons, respectively (Table 2). Seven components, trans-verbenol, trans-verbenyl acetate, 2-ethylidene-6-methyl-3,5-heptadienal, longifolene, cis-beta-farnesene, selina-3,7(11)-diene and caryophyllenyl alcohol, were not detected in the EO of Indian blackberry leaves harvested in the winter. Thus, the Indian blackberry leaves harvested during the spring season produced a higher spectrum of chemical components in its EO. Terpinolene, L-alpha-terpineol and bicyclo [5.2.0] nonane, 2-methylene-4,8,8- trimethyl-4-vinyl- were major components of both essential oils. α-Terpineol has previously been identified in *S. cumini* EO [32].

Terpinolene is a monocyclic terpene hydrocarbon with a turpentine-like odor. It has a role as a plant metabolite, an insect repellent, and as a sedative. Alloocimene is an acyclic monoterpenoid that has a pleasant odor and is used in the perfume industry. Carveol is widely used as an antioxidant and anti-inflammatory and in traditional medicine. Limonene is used as a flavorant in foods and in fragrances. Bornyl acetate is the acetate ester of borneol. It is used as a food additive for its flavor and aroma. Bicyclo [5.2.0] nonane, 2-methylene-4,8,8-trimethyl-4-vinyl- is a sesquiterpene and highly bioactive compound. Aromadendrin is used topically in the treatment of various skin problems.

### 3.2. Fractionation of Essential Oil

EO vacuum fractional distillation separates compounds based on differences in volatility, physical and chemical features, temperature, and the degree of vacuum applied. It is impossible to separate EO components at atmospheric pressure as they usually boil and evaporate as a single azeotropic mixture. However, under reduced pressure, EO can be easily separated into different components. Indian blackberry leaf EO was fractionated under high vacuum (−760 mmHG) into three fractions (F1, F2, F3) and the residue (R) based on their boiling range (Table 3). F1 had the lowest boiling point range (39–41.6 °C) followed by F2 isolated with a boiling range of 40–59 °C, and F3, which had a boiling range 37–60 °C after removal of F2. The EO was never heated above 60 °C to avoid thermal degradation of compounds. The residue consisted of the remaining components that had boiling points above 60 °C.

Indian blackberry leaf EO was also processed by slow cooling to −50 °C to produce two fractions including the non-crystalline fraction (NCF) and the crystalline fraction (CF) (Table 3). This process was adopted to avoid the possibility of any thermal degradation that could result during heating. Most terpenes are thermally unstable and undergo decomposition and oxidation at high temperatures in the presence of oxygen or light [33]. Therefore, a high vacuum was preferred during the present study to reduce the temperature of vaporization of the volatile mixture. Studies on the fractional distillation of EO are rare although this process is very common in the petrochemical industry [8].

### 3.3. Antioxidant Activities

**Figure 2:**
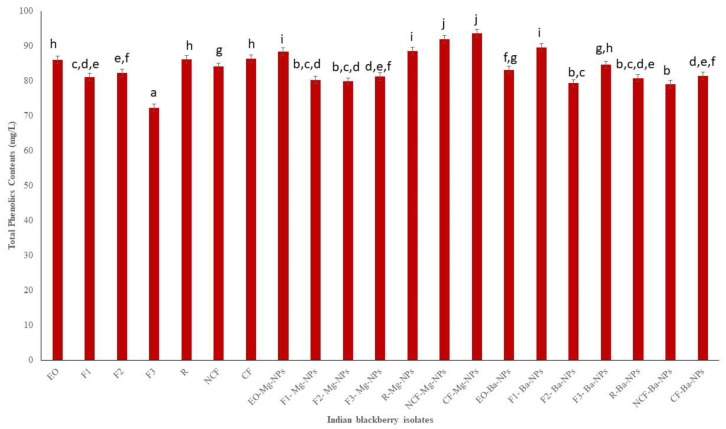
Total phenolics contents (TPC) of Indian blackberry isolates. Different letters on the bar of the graph indicate the significantly different (*p* < 0.05) within essential oil, fractions, and nanoparticles.

Based on biological and chemical mechanisms, antioxidant assays have been used to determine the antioxidant potential of plant samples to obtain semiquantitative and quantitative data. The antioxidant potential of Indian blackberry leaf EO and EO fractions and nanoparticles was determined by measuring total phenolics contents (TPC), total flavonoids contents (TFC), and 1,1-diphenyl-2-picryl-hydrazyl (DPPH) radical scavenging activity. Phenolics and flavonoids have redox properties that contribute to the antioxidant activity of plant extracts and EO. The presence of the hydroxyl groups in phenolics play a vital role in facilitating free radical scavenging, and there is often a correlation of total phenolic content with free radical scavenging activity in plant extracts [34]. This occurs because by increasing the phenolic moiety, resonance in the molecule also increases which decreases the energy of the molecule and enhances its stability [35]. The TPC of Indian blackberry EO, EO fractions, and nanoparticles ranged from 72.32 mg GAE/L to 93.57 mg GAE/L (Figure 2). The highest TPC was in the crystalline fraction Mg nanoparticles (CF-Mg-NPs). Thus, the slow cooling process was effective in isolating a fraction with higher phenolic contents while avoiding thermal degradation and polymerization reactions that usually result with heating.

**Figure 3:**
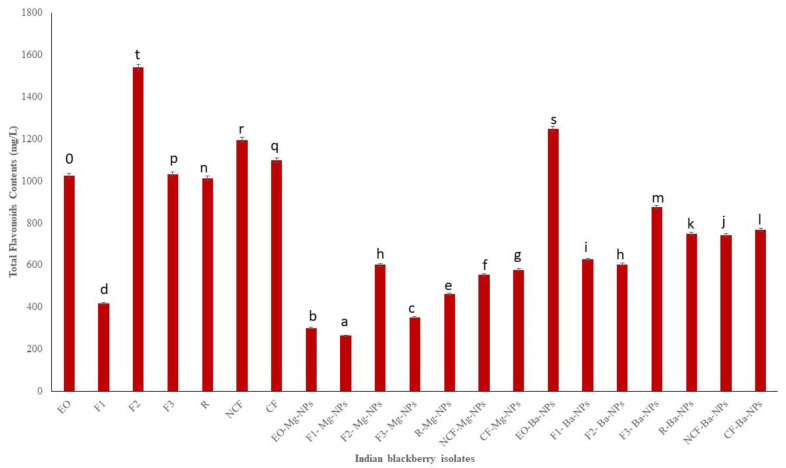
Total flavonoids contents (TFC) of Indian blackberry isolates. Different letters on the bar of the graph indicate the significantly different (*p* < 0.05) within essential oil, fractions, and nanoparticles.

Flavonoids, colloquially known as vitamin P [36], are another class of secondary metabolites that typically provide yellow and other colored pigments to plants. Flavonoid metabolites are readily absorbed by humans [37] and display anti-inflammatory and anti-cancer activities [38,39]. TFC measured in the present study ranged from 265 to 1540.55 mg/L of catechin equivalent (Figure 3). Fraction 2 (F2) had the highest TFC indicating that the high vacuum fractional distillation could be used to concentrate EO flavonoids.

**Figure 4:**
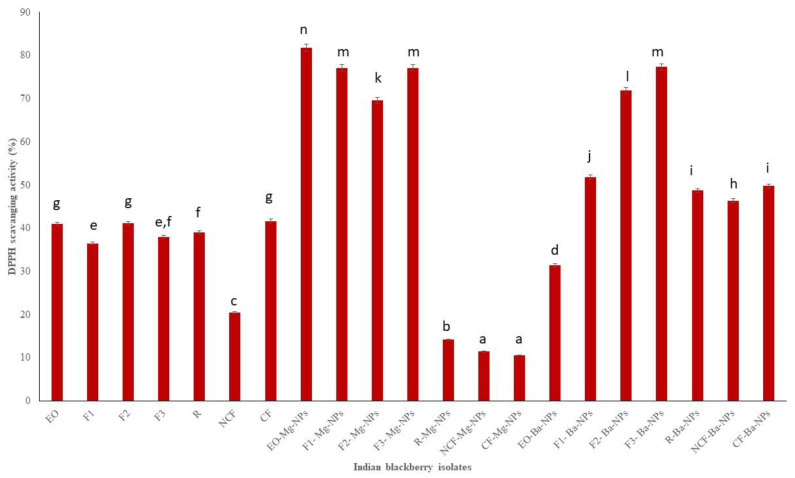
DPPH scavenging activity of Indian blackberry isolates. Different letters on the bar of the graph indicate the significantly different (*p* < 0.05) within essential oil, fractions, and nanoparticles.

DPPH is a stable free radical that is used in antioxidant studies [40]. Indian blackberry EO Mg nano particles had the highest DPPH scavenging activity (Figure 4). Overall, F1 had the lowest TPC, TFC, and DPPH scavenging activities. Fraction 1 (F1) had the lowest boiling point and therefore contained the most volatile components. TPC, TFC, and DPPH scavenging activities are usually higher with higher molecular compounds that can scavenge free radicals. It is also very clear from the results that Mg nanoparticles had higher antioxidant activities than Ba nanoparticles

The antioxidant activities of pure essential oils and its nano particle were tested. The obtained results of TPC, TFC, and DPPH of pure essential oil were compared with maximum and minimum values. The obtained results are shown on the Figure 2, Figure 3 and Figure 4 clearly depicted that the TPC, TFC, and DPPH values of essential oil, fractions and nanoparticles were significantly different.

## 4. Conclusions

The following conclusions were drawn from the present study: first, the EO yields from Indian blackberry leaves were higher in the spring season as compared to winter season. Indian blackberry leaves harvested during spring also produced a slightly broader spectrum of chemical components. Both vacuum fractional distillation and slow cooling under vacuum can be used to separate and concentrate antioxidant compounds of interest. The EO, F2, and CF had the highest antioxidant activities when measured as neat fractions. Magnesium nanoencapsulation significantly improved free radical scavenging activities of the EO and F1–F3, while Ba nanoencapsulation improved the activity of F1–F3 as well as the R, NCF, and CF.

## Figures and Tables

**Table 1:** Sample codes and treatment descriptions.

Sr. No.	Code	Description
1	EO	Indian blackberry essential oil
2	F1	Indian blackberry EO fraction 1 isolated through high vacuum fractional distillation
3	F2	Indian blackberry EO fraction 2 isolated through high vacuum fractional distillation
4	F3	Indian blackberry EO fraction 3 isolated through high vacuum fractional distillation
5	R	Indian blackberry EO residue left after high vacuum fractional distillation
6	NCF	Non-crystalline fraction of EO (NCF) obtained by slow cooling to −50 °C
7	CF	Crystalline fraction of EO (CF) obtained by slow cooling to −50 °C
8	EO-Mg-NPs	EO Mg nanoparticles
9	F1-Mg-NPs	Fraction 1 Mg nanoparticles
10	F2-Mg-NPs	Fraction 2 Mg nanoparticles
11	F3-Mg-NPs	Fraction 3 Mg nanoparticles
12	R-Mg-NPs	Residue Mg nanoparticles
13	NCF-Mg-NPs	Non-crystalline fraction of EO (NCF) Mg nanoparticles
14	CF-Mg-NPs	Crystalline fraction of EO (CF) Mg nanoparticles
15	EO-Ba-NPs	EO Ba nanoparticles
16	F1-Ba-NPs	Fraction 1 Ba nanoparticles
17	F2-Ba-NPs	Fraction 2 Ba nanoparticles
18	F3-Ba-NPs	Fraction 3 Ba nanoparticles
19	R-Ba-NPs	Residue Ba nanoparticles
20	NCF-Ba-NPs	Non-crystalline fraction of EO (NCF) Ba nanoparticles
21	CF-Ba-NPs	Crystalline fraction of EO (CF) Ba nanoparticles

**Table 2:** The GC-MS analysis of Indian blackberry leaves essential oils.

Sr. No.	Compound Name	Spring Season	Winter Season
		Compounds (%)	Compounds (%)
1.	gamma-Terpinene	0.70	6.45
2.	Citral	0.45	0.55
3.	Terpinolene	14.03	11.10
4.	Linalool	4.15	0.53
5.	Fenchol	1.65	0.42
6.	Alloocimene	4.47	2.84
7.	Cyclohexene, 1-methyl-3-(formyl methyl)-	0.36	4.58
8.	Carveol	2.81	16.50
9.	trans-Verbenol	0.55	Nd
10.	Sabinene hydrate	0.33	0.32
11.	endo-Borneol	0.29	0.32
12.	Terpinen-4-ol	1.67	0.97
13.	L-alpha-Terpineol	8.54	4.37
14.	Methyl salicylate	1.02	0.28
15.	(3E,5E)-2,6-Dimethylocta-3,5,7-trien-2-ol	0.64	4.71
16.	Bicyclo [2.2.1] heptan-2-ol, 1,3,3-trimethyl-, acetate, (1S-exo)-	3.40	0.97
17.	2-Isopropylidene-3-methylhexa-3,5-dienal	0.15	2.19
18.	p-Mentha-1(7),8-dien-2-ol	0.23	0.26
19.	Bornyl acetate	5.16	0.13
20.	Trans-Verbenyl acetate	0.53	Nd
21.	Ethylene Carbonate	0.72	9.12
22.	trans-Verbenol	0.15	0.70
23.	Ylangene	0.16	0.36
24.	alpha-Copaene	4.36	0.32
25.	(3E,5E)-2,6-Dimethylocta-3,5,7-trien-2-ol	1.89	0.72
26.	(+)-Isopiperitenone	3.70	2.13
27.	alpha-Longipinene	0.33	1.66
28.	Bicyclo [5.2.0] nonane, 2-methylene-4,8,8- trimethyl-4-vinyl-	12.53	2.55
29.	3,5-Heptadienal, 2-ethylidene-6-methyl-	0.32	Nd
30.	Longifolene	0.19	Nd
31.	Aromandendrene	1.11	8.60
32.	1,4,7,-Cycloundecatriene, 1,5,9,9-tetramethyl-, Z,Z,Z-	6.07	0.40
33.	cis-.beta.-Farnesene	3.77	Nd
34.	Octahydronaphthalene	1.64	0.69
35.	gamma.-Muurolene	0.98	0.26
36.	alpha.-Muurolene	0.19	0.74
37.	beta-Selinene	0.71	0.83
38.	Naphthalene, 1,2,3,5,6,7,8,8a-octahydro-1,8a- dimethyl-7-(1-methylethenyl)-, [1R- (1.alpha.,7.beta.,8a.alpha.)]-	1.52	0.26
39.	gamma.-Maaliene	1.14	0.95
40.	alpha.-Muurolene	0.68	1.07
41.	gamma.-Muurolene	0.90	3.38
42.	Cadinene	1.82	2.12
43.	Naphthalene	0.17	1.51
44.	Selina-3,7(11)-diene	0.28	Nd
45.	Caryophyllenyl alcohol	0.16	Nd
46.	beta-Eudesmol	1.26	1.16
47.	Viridiflorol	0.20	0.32
48.	Humulene epoxideII	0.41	0.26
49.	1-Oxaspiro [2.5] octane, 5,5-dimethyl-4-(3- methyl-1,3-butadienyl)-	0.35	0.24
50.	tau-Cadinol	0.23	0.26
51.	d-Ledol	0.47	0.54
52.	beta-Acoradienol	0.19	0.33

**Table 3:** Isolated fractions of Indian blackberry essential oil.

Fractions	Isolation Temperature (°C)	Percentage of Fractions (%)
F1	39–41.6	14.10 ± 0.21 ^a^
F2	40–59	25.71 ± 0.13 ^c^
F3	37–60	18.57 ± 0.30 ^b^
R	>60	39.13 ± 0.44 ^e^
NCF	−41	71.30 ± 0.35 ^f^
CF	<−41	28.70 ± 0.49 ^d^

^a,b,c,d,e,f^ values with different letters in the same column are significantly different (*p* < 0.05).

## Data Availability

Not Applicable.
